# A Three-Dimensional Co-Culture Model for Rheumatoid Arthritis Pannus Tissue

**DOI:** 10.3389/fbioe.2021.764212

**Published:** 2021-11-12

**Authors:** Jietao Lin, Antonia RuJia Sun, Jian Li, Tianying Yuan, Wenxiang Cheng, Liqing Ke, Jianhai Chen, Wei Sun, Shengli Mi, Peng Zhang

**Affiliations:** ^1^ Center for Translational Medicine Research and Development, Shenzhen Institute of Advanced Technology, Chinese Academy of Science, Shenzhen, China; ^2^ University of Chinese Academy of Sciences, Beijing, China; ^3^ Shenzhen Engineering Research Center for Medical Bioactive Materials, Shenzhen, China; ^4^ Biomanufacturing Center, Department of Mechanical Engineering, Tsinghua University, Beijing, China

**Keywords:** rheumatoid arthritis, 3D bioprinting, tissue engineering, pannus tissue model, drug screening

## Abstract

Three-dimensional (3D) co-culture models have closer physiological cell composition and behavior than traditional 2D culture. They exhibit pharmacological effects like *in vivo* responses, and therefore serve as a high-throughput drug screening model to evaluate drug efficacy and safety *in vitro*. In this study, we created a 3D co-culture environment to mimic pathological characteristics of rheumatoid arthritis (RA) pannus tissue. 3D scaffold was constructed by bioprinting technology with synovial fibroblasts (MH7A), vascular endothelial cells (EA.hy 926) and gelatin/alginate hydrogels. Cell viability was observed during 7-day culture and the proliferation rate of co-culture cells showed a stable increase stage. Cell-cell interactions were evaluated in the 3D printed scaffold and we found that spheroid size increased with time. TNF-α stimulated MH7A and EA.hy 926 in 3D pannus model showed higher vascular endothelial growth factor (VEGF) and angiopoietin (ANG) protein expression over time. For drug validation, methotrexate (MTX) was used to examine inhibition effects of angiogenesis in 3D pannus co-culture model. In conclusion, this 3D co-culture pannus model with biological characteristics may help the development of anti-RA drug research.

## Introduction

Joint is a dynamic tissue that supports us to move, but it may suffer destruction of bone and cartilage because of arthritis like RA. Due to the genetic factor or immune system disorder, synovial membrane in RA patients presents abnormal proliferation of synovial cells and migration of inflammatory cells (Deane et al., 2017). Synovial joint is usually rich in blood vessels, which is a unique manifestation of RA. New vessels and hyperplastic fibrous tissue contribute to angiogenic disorders and form a complex vascular tissue called pannus (Veale et al., 2017). Angiogenesis not only provides more means for the spread of inflammatory cytokines and the infiltration of leukocyte but aggravate the formation of pannus (Maruotti et al., 2006; Elshabrawy et al., 2015). RA pannus is an aggressive and invasive tissue with massive leukocyte infiltration, proliferative synovial membranes and neovascularization, which is directly responsible for cartilage destruction and bone erosion (Lee and Weinblatt, 2001). The development of pannus is highly relevant to the growth factors, pro-inflammatory cytokines and chemokines. Growth factors, such as vascular endothelial growth factor (VEGF) and basic fibroblast growth factor (bFGF) are described as the key regulators in proliferation, migration and vascular formation. Pro-inflammatory cytokines like tumor necrosis factor (TNF)-α, interleukin (IL)-6, which provide inflammatory conditions in RA synovium, have direct and indirect effects on other cell types to produce pro-angiogenic factors (Semerano et al., 2011).

In recent years, RA have become the most common form of inflammatory arthritis. Patients need to rely on drugs for control as it is an incurable disease (Doan and Massarotti, 2005; Yu et al., 2018). Inhibition of angiogenesis can be a helpful strategy for early prevention and treatment of RA (Veale and Fearon, 2006; MacDonald et al., 2018; Balogh et al., 2019). However, RA drug testing has low accuracy and drug development cycle is long. Although animal model is the most effective way to study RA drugs before clinical trials, ethics and experimental accuracy limits rapid and efficient evaluation of drug safety and efficacy (Li and Izpisua Belmonte, 2019). To overcome these difficulties, co-culture models are often used to mimic physiological environment of pannus for RA study and anti-RA drug screening (D’andrea et al., 1998; Kasama et al., 2001; Nozaki et al., 2007; Chu et al., 2018; Gou et al., 2018). IBOLD et al. developed a 3D pannus model *in vitro* as a high-throughput screening assay. Chondrocytes from porcine donors were isolated and seeded them into wells to form extracellular matrix (ECM). After 14 days, it would be coated with human synovial fibroblasts. They found that intercellular communication between these 2 cell types occurs both through gap junctions and ATP-mediated paracrine stimulation. (D’andrea et al., 1998). In the co-culture model of chondrocytes and synovial cells, D’ANDREA et al. found that the Ca + signal between these 2 cell types can be affected by 18α-glycyrrhetinic acid, suggesting they have communication in pannus tissue (Nozaki et al., 2007). Monocytes or polymorphonuclear neutrophils (PMNs) were seeded onto fibroblasts and Kasama et al. found that the expression of VEGF in co-culture groups are higher than synovial fibroblasts, monocytes or PMNs alone groups, which means VEGF expression in pannus can be also regulated by the interaction of synovial fibroblasts and activated leukocytes (Chu et al., 2018). Nozaki et al. isolated pannus tissue from RA patients and the inflammatory cells including macrophages, T cells and fibroblasts. They collected these cell types without enzyme digestion and found that inflammatory cells could develop into pannus-like tissue spontaneously *in vitro*. This pannus model continuously secreted MMP-9 and TNF-α, IL-8 and M-CSF, which related with osteoclastogenesis (Gou et al., 2018). Although these studies revealed useful characteristics of 3D pannus models *in vitro*, it is still difficult to construct a long lasting and strong repeatability pannus model to test anti-RA drugs due to the limitations of fabrication techniques.

**FIGURE 1:**
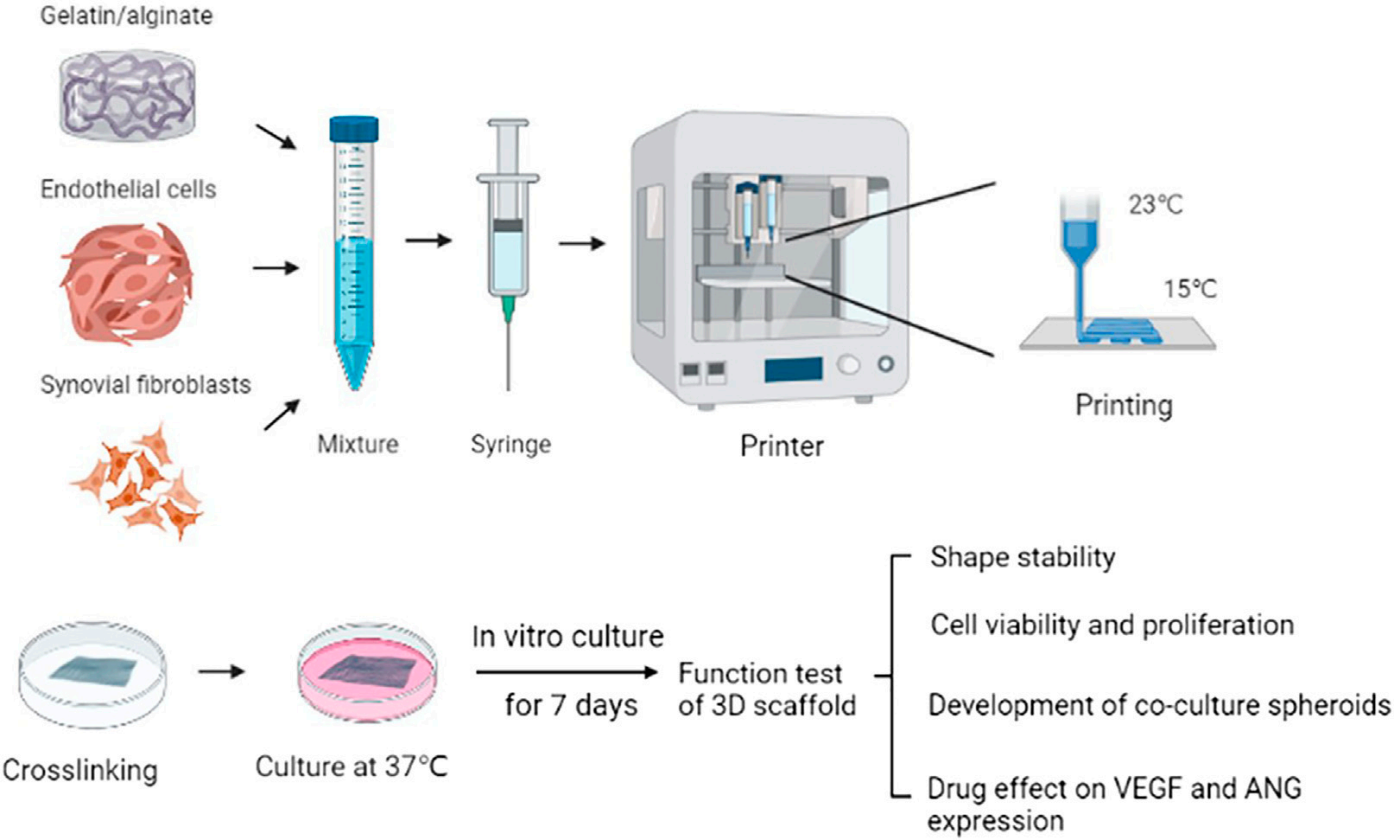
Schematic of the 3D scaffold printing process. Fabrication of pannus mimic with gelatin /alginate and EA.hy 926/MH7A cells.

Recent advances in 3D fabrication technology have allowed direct assembly of cells and biocompatible materials to form *in vitro* cellular models for artificial organ regenerations, the study of disease mechanisms and drug screening. This promising technique has the advantages of accurate control of cell distribution, high simulation of physiological microenvironments and cost-effectiveness, which is suitable for constructing complex 3D *in vitro* models (Mandrycky et al., 2016; Ma et al., 2018; Ong et al., 2018; Zhu et al., 2020). Therefore, 3D printing has been applied in the establishments of disease pathogenesis and drug screening model in hepatocellular carcinoma (Sun et al., 2020; Xie et al., 2021), breast cancer (Swaminathan et al., 2019; Lv et al., 2021), cervical tumor (Zhao et al., 2014; Pang et al., 2018), bladder cancer (Kim et al., 2019), and neurodegenerative diseases (Thomas and Willerth, 2017). To apply the potential value of 3D printing on anti-RA drug research, in this paper we constructed the *in vitro* pannus model by 3D printing of endothelial cells (EA.hy 926)/Synovial fibroblasts (MH7A) and gelatin/alginate and characterized its biological function. To our knowledge, RA synovial tissue fibroblasts produce pro-angiogenic growth factors, cytokines under the induction of inflammatory mediators or hypoxia. Under the condition of pro-angiogenic and inflammatory factors, endothelial cells therefore promote cell proliferation, migration and tube formation (Szekanecz et al., 2005; Elshabrawy et al., 2015; Alam et al., 2017; Croft et al., 2019). Both MH7A cell line (synovial fibroblasts) and EA.hy 926 cell line (endothelial cells) are widely used to be the cell model in RA research as they are considered valuable in preclinical trials (Komorowski et al., 2006; Cheng et al., 2019; Qu et al., 2019; Kong et al., 2020). In addition, we used gelatin/alginate as they can mimics ECM to provide the cells a better natural microenvironment. They show good biocompatibility and good molding effect when building 3D biological scaffolds, and these structures could have long retention time (Sun et al., 2020; Lv et al., 2021). The schematic of 3D pannus scaffold printing process has been showed in Figure 1. Biological characterization of 3D printed pannus models on calcium cross-linking toxicity, cell proliferation, cell survival, cell morphology and VEGF and Angiopoietin (ANG) protein expression will be evaluated. Our findings may offer a basic view of 3D printed pannus model in drug screening application.

## Materials and Methods

### Cell Culture

Human endothelial cells (EA.hy 926) were generously given by professor Qin’s Laboratory and the cells were cultured in Dulbecco Modified Eagle Medium (DMEM) with 4.5 g/L glucose, supplemented with 10% fetal bovine serum (Gibco, Thermo Fisher Scientific) and 1% penicillin/streptomycin (Sigma-Aldrich, MO, United States). Human synovial fibroblasts (MH7A) were purchased from the Riken Cell Bank (Tsukuba, Japan). The cells were maintained in Roswell Park Memorial Institute (RPMI) cultivation medium (Hyclone, Thermo Fisher Scientific, Wilmington, DE, United States) plus 10% fetal bovine serum (Gibco, Thermo Fisher Scientific) and 1% penicillin/streptomycin (Sigma-Aldrich). For TNF-α co-culture model, EA.hy 926 and MH7A were pretreated with 20 ng/ml TNF-α for 6 h before printing. All culture experiments are under the condition of humidified air with 5% CO_2_ in 37°C.

### Bioink Preparation

Gelatin/alginate was purchased from Sunp Biotech (Beijing, China). The lyophilized powder was dissolved in DMEM medium at 56°C. The mixed ink should be pasteurized by 70°C for 30 min and 4°C for 10 min three times at constant temperature water bath and stored at 4°C until use. Before the experiment, the ink was kept at 37°C for 2 h. For co-culture bioink, 2.5×10^6^ cells of each EA.hy 926 and MH7A were resuspended in DMEM medium and homogeneously mixed with gelatin/alginate ink at a volume ratio at 1:4, resulting in a final cells density of 10^6^ cells/ml.

### 3D Scaffold Formation and Culture

3D Cell Printer (SPP1603, SUNP, China) were used to fabricate all the 3D scaffold models. The temperature of the nozzle and printing bed were 23 and 15°C, respectively. 25G needle was chosen and the scanning speed was controlled at 3 mm/s. The models were printed in an eight-layered square grid pattern with the size of 10 × 10 mm cross sectional area and 2.4 mm thickness. The inks were loaded into 3 ml printing syringe and precooled at the printing chamber for 10 min. Preprinted on the 35 mm petri dishes and ensured that the bioink was smoothly extruded. After printing, hydrogel scaffolds were immersed in CaCl_2_ solution for 5 min for crosslinking with alginate, providing better strength to the scaffolds. All scaffolds were gently blown with a pipette to remove bubbles. Then the scaffolds were washed with sterile physiologic saline once and finally cultured in DMEM medium. The scaffolds were crosslinked and the medium was changed every 3 days.

### Calcium Cross-Linking Toxicity

EA.hy 926, MH7A and co-culture mixed cells were seeded into 96-well culture plates (3,000 cells/well) for 24 h. The cells were stimulated by 3% CaCl_2_ solution for 5 min and washed by physiologic saline, and then they were cultured in DMEM medium for another 24 or 48 h. To evaluate cellular metabolic activity, Cell counting kit-8 (CCK-8, Dojindo, Japan) were added into each well at the volume of 10% of the total, protected against exposure to light. After 3 h incubation at 37°C, fluorescence of the culture medium was detected by microplate reader (PerkinElmer, Waltham, MA) at 450 nm. The data was then normalized to the standard and calculated cell viability.

### Cell Proliferation Analysis

Cell proliferation in printed scaffolds was studied using CCK-8 on cultured days 1, 3, 5 and 7. Cells were incubated in a mixture of culture medium and CCK-8 for 2 h. The values of fluorescence at 450 nm were compared among different printed groups.

### Cell Survival

Cell survival test in 3D scaffolds was carried out on day 1, 3, 5, 7 after printing. Fluorescent Live/Dead assay (C2015M, Beyotime, China) was used according to the instruction manual. Briefly, medium was removed and the scaffolds were then washed twice with phosphate buffer solution (PBS). Subsequently, Calcein-AM and propidium iodide (PI) was mixed with detection buffer at the dilution ratio of 1:1,000 and 1.5:1,000, respectively. The cell laden scaffolds were incubated at 37°C for 30 min in dark, then washed three times with PBS. Calcein-AM marks viable cells green and propidium iodide (PI) shows dead cells red. Images were obtained from fluorescence microscopy (Leica, Germany).

### Cell Morphology Imaging and Analysis

The scaffold shapes were taken with camera (Supplementary Figure S1). Two dimensional cell morphology of EA.hy 926 and MH7A was captured using inverted optical microscope (Carl Zeiss, Germany) at cell density of 80%. Three-dimensional scaffold was examined by using fluorescence microscope (Leica, Germany) on cultured days 1, 3, 5 and 7. The images were taken in three random fields at 100× magnification. Cell diameters were measured by Image J (NIH, United States) software and analyzed by Origin (Originlab, United States).

### Enzyme-Linked Immunosorbent Assay

The experiment was divided into four groups: blank (no cell) group, co-culture group, TNF-α pannus model group and TNF-α pannus treated by 100 nM MTX for 24 h group. The concentrations of VEGF and ANG protein in the culture medium of 3D scaffold were detected on day 1, 3, 5, 7. Secretory cytokines were examined using corresponding commercial ELISA kits (R&D Systems, United States). A standard curve was constructed for each assay according to the manufacturer’s instructions. The cytokine concentrations of each sample were calculated on the basis of the standard curve.

### Statistical Analysis

All the data were presented as mean ± SD. Statistical significance was evaluated by Students T Test. Differences were considered to be significant for *p* < 0.05. *p < 0.05; **p < 0.01; ***p < 0.001. Each experiment was performed in triplicate (*n* = 3) on at least independent three samples (*N* ≥ 3).

## Results

### Calcium Toxicity on Cells in 2D Planar Culture

**FIGURE 2:**
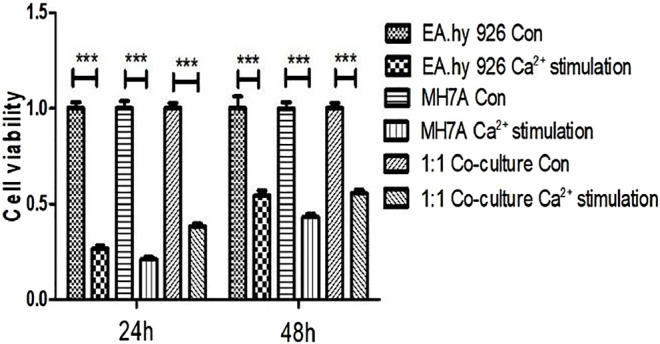
Cytotoxicity of calcium on EA.hy 926 and MH7A and 1:1 co-culture mixture in 2D planar. Cells were treated with CaCl2 solution for 5 min and washed with physiologic saline once. After 24 and 48 h, their viability was determined using CCK-8 assay (mean ± SD, ^*^ *p* < 0.05; ^**^ *p* < 0.01; ^***^ *p* < 0.001, t-test). Con = no calcium stimulation; SD = standard deviation.

To test the cytotoxic effect of calcium on EA.hy 926 and MH7A and 1:1 co-culture mixture, CCK-8 was used to determine the cell viability. As is shown in Figure 2, after stimulating by calcium for 5 min and cultured in DMEM for 24 h, The percentage of EA.hy 926 cell viability decreased to 26.53 ± 4.34, but it rose to 54.49 ± 6.17 at 48 h MH7A cells also had the same trend, 21.05 ± 3.30 percent at 24 h and 43.01 ± 4.24 percent at 48 h, respectively. In terms of 1:1 co-culture mixture, they performed 38.29 ± 3.71 at 24 h and 55.62 ± 4.17 at 48 h.

### Cell Distribution and Viability in 3D Scaffolds

**FIGURE 3:**
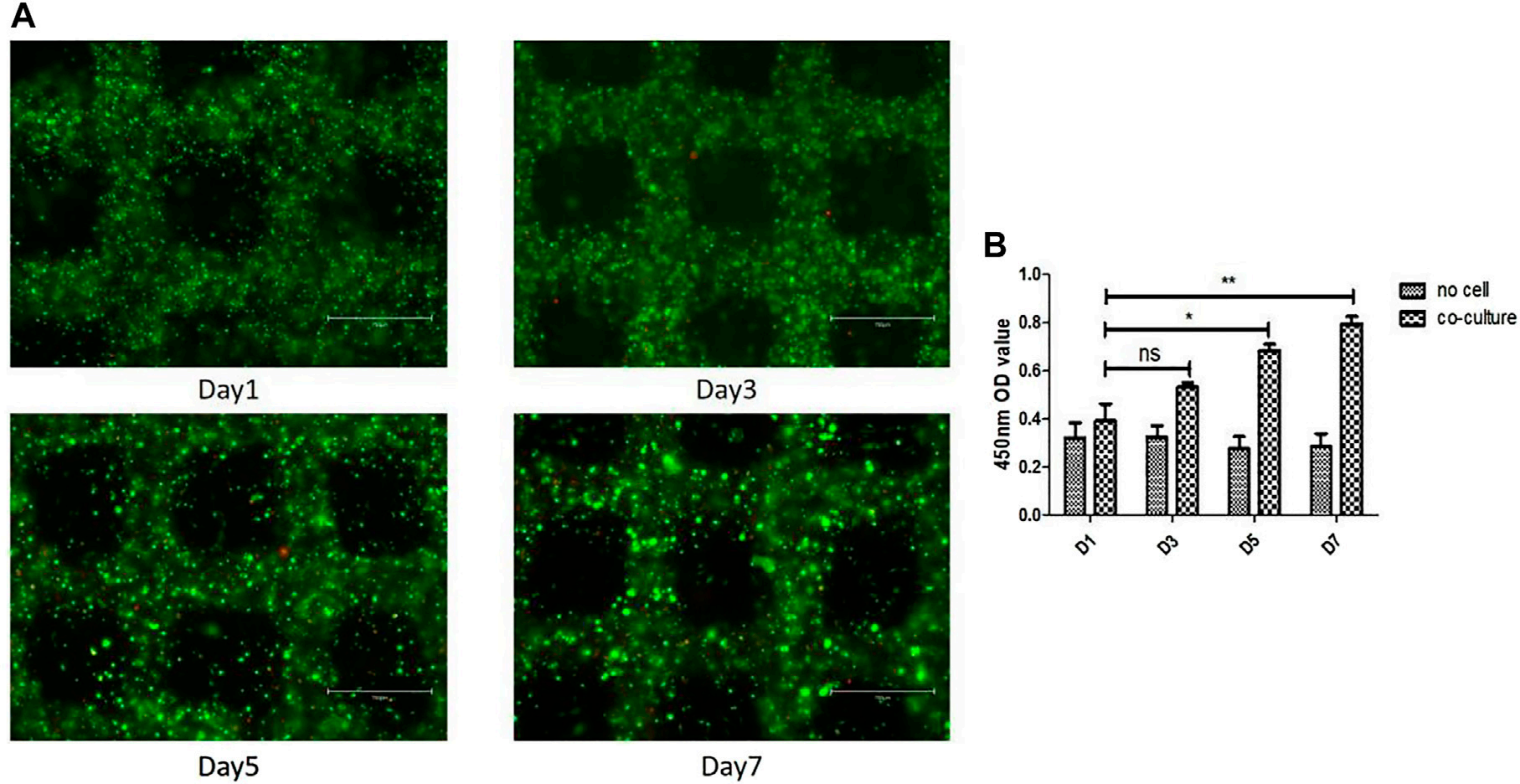
Cell survival and proliferation in the 3D cell laden pannus tissue model. **(A)** Cell survival at different time points after printing. Live and dead cells were labelled with calcein-AM (green) and PI (red), respectively. Scale bar, 750 μm. **(B)** Proliferation rates of cells in 3D co-culture cell laden scaffolds at day 1, 3, 5, and 7.

To determine cell distribution and survival in 3D gelatin/alginate/EA.hy 926/MH7A model, we used calcein-AM/PI staining assay to analyze live/dead cells on day 1, 3, 5 and 7. As we can see, the cells were evenly distributed in gelatin/alginate scaffold. Cell viability was stable about 80% during the *in vitro* culture of EA.hy 926/MH7A in 3D scaffold (Figure 3A). The cellular proliferation in 3D scaffolds was detected using CCK-8 kit on the same time. Figure 3B demonstrates that compared with day 1, cells had 1.36-fold proliferation on day 3, 1.75-fold proliferation on day 5, and 2.03-fold proliferation on day 7. There were significant differences between day 1 (0.39 ± 0.12) and day 5 (0.68 ± 0.05) and 7 (0.79 ± 0.05). Overall, the proliferation rate of co-culture cells had a stable increase stage from day 1 to day 7.

### Development of Co-Culture Spheroids Within Cell Laden Scaffold

**FIGURE 4:**
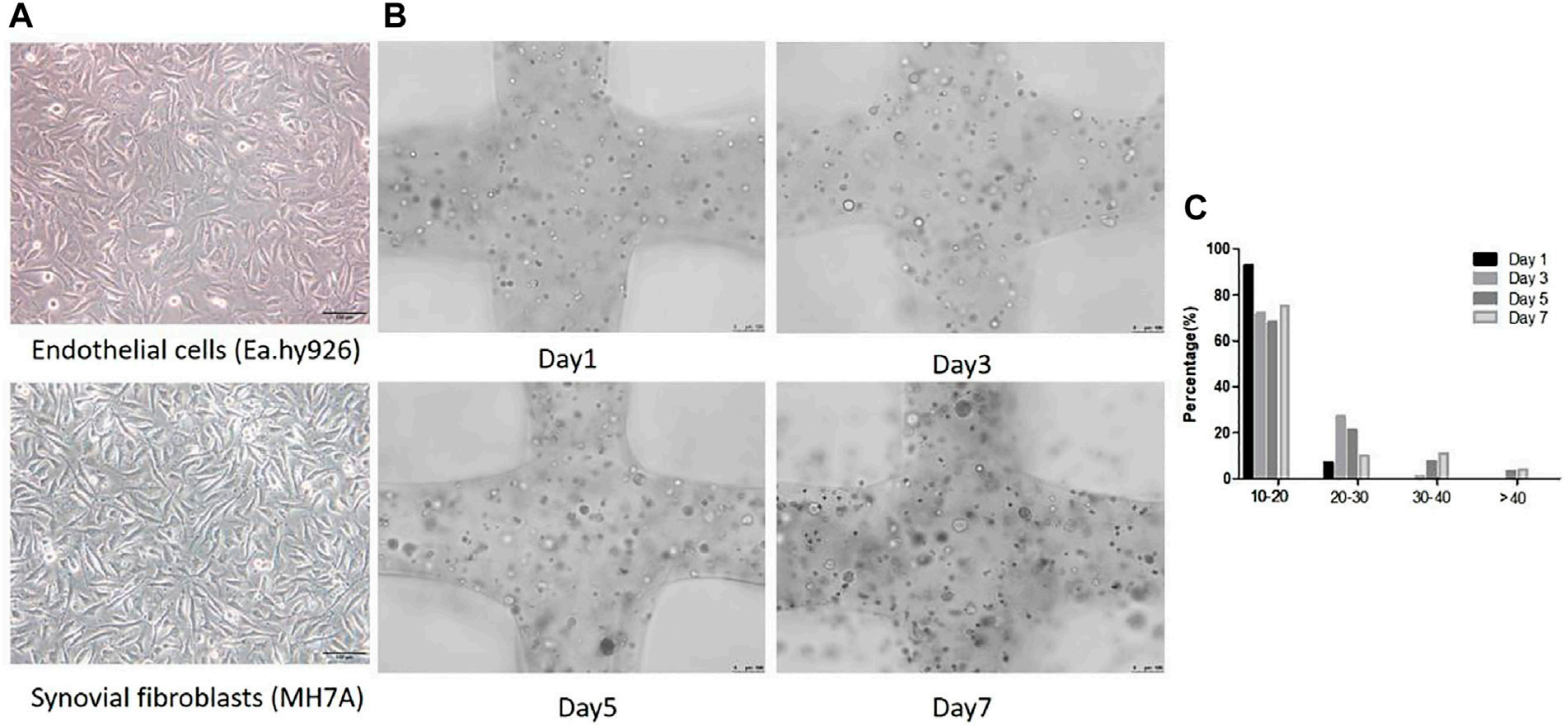
Cellular morphological differences between 2D planar culture and 3D scaffolds. **(A)** EA.hy 926 and MH7A cells morphology in 2D planar culture. EA.hy 926 cells look epithelioid and MH7A cells are epithelioid and polygonal. Scale bar, 100 μm. **(B)** 3D Gelatin /alginate/EA.hy 926/MH7A scaffolds observed by a fluorescence microscope on culture days 1, 3, 5, and 7. Scale bar, 100 μm. Black arrows indicate cells and cellular spheroids in 3D scaffolds. **(C)** Distribution of spheroid diameter in 3D cell laden scaffolds on day 1, 3, 5, and 7.

Inverted optical microscope was used to observe the cell morphology in 2D planar culture. EA.hy 926 shows epithelioid morphology and MH7A shows epithelioid and polygonal morphology (Figure 4A). The cells and cellular distribution pattern in printed scaffolds were characterized using fluorescence microscope on day 1, 3, 5, 7. Compared with 2D planar culture, cells turned to be spheroids within 3D scaffolds, and they were observed to form larger spheroids after 3 days of bioprinting. The spheroid size increased over time. Figure 4B shows spheroid distribution in the 3D co-culture cell laden scaffold. At day 1, 7.09% of the spheroids were at the diameter range of 20–30 μm. At day 3, the size between 20–30 μm was up to 28%. The percentages of over 30 μm at day 5 and day 7 were 10.65 and 14.73%, respectively (Figure 4C).

### Effect of 3D Engineered Scaffold on VEGF and ANG Expression

**FIGURE 5:**
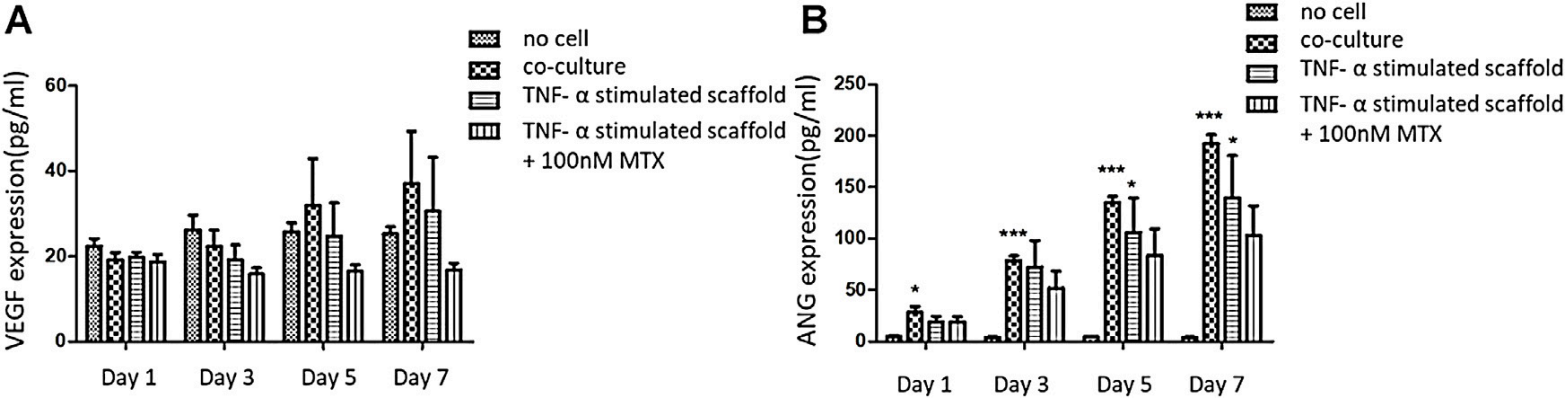
Concentration of VEGF and ANG secreted protein in the supernatant of the three-dimensional scaffold on Day 1, 3, 5, and 7. **(A)** A. VEGF secreted protein concentration (pg/ml); **(B)** ANG secreted protein concentration (pg/ml).

The experiment was divided into four groups: blank (no cell) group, co-culture group, TNF-α pannus model group and TNF-α pannus treated by MTX group. The culture medium of the three-dimensional scaffold was collected on day 1, 3, 5, 7. Figure 5A shows that the cell co-culture scaffold and pannus scaffold secreted more VEGF protein on day 5 and 7 compared with that on the first day, but there was no significant difference compared with the no cell group on the same day (*p* > 0.05). Figure 5B illustrates the content of ANG secreted protein in the co-culture scaffold on day 1 was different from that of the no cell group (*p* < 0.05), and there was a significant difference from day 3 to day 7 (*p* < 0.001). On the day 7, the ANG protein concentration of the TNF-α pannus model group was different from that of the blank group (*p* < 0.05). Although the ANG concentration decreased in the pannus MTX group, there was no significant difference when compared with that in TNF-α pannus group.

## Discussion

RA is a chronic and systemic autoimmune disease, and immune dysregulation occurs earlier than joint inflammation. Current medications for RA are glucocorticoids (GCs), nonsteroidal anti-inflammatory drugs (NSAIDs), disease-modifying antirheumatic drugs (DMARDs) and biological therapies, which provide clinically meaningful pain relief and control inflammation in patients. However, several side effects, including cytopenia, psoriasis, lung disease and liver damage, have been proved in treatments with these drugs (Burmester and Pope, 2017; Abbasi et al., 2019). Thus, the discovery of a safe and effective drug for RA treatment remains a crucial challenge.

Over the last 2 decades, angiogenesis has been reported to play an important role in the deterioration of RA. RA pannus is an aggressive and invasive tissue with rich proinflammatory cytokines like TNF-α, IL-1β and IL-6, which is directly responsible for cartilage destruction and bone erosion (Lee and Weinblatt, 2001; MacDonald et al., 2018). Numerous researchers have studied on inhibition of angiogenesis in RA (Zhang et al., 2017; Yang et al., 2018; Li et al., 2019; Zhai et al., 2019). In order to screen anti-RA drugs in a more accurate and efficient way, here we developed a 3D printed model to mimic the microenvironment of RA pannus. In terms of cell composition, we chose vascular endothelial cells and synovial fibroblast. Since EA.hy 926 and MH7A cells are widely used in drug discovery in RA and they are cell lines, we chose them to ensure the effectiveness and repeatability of our model. However, single cell model failed to offer a complex microenvironment to evaluate cellular response to drugs accurately (Sun et al., 2019). In the microenvironment, we added inflammatory factors (TNF-α) to simulate the inflammatory microenvironment. Compared to monolayer cell culture, 3D printing model is close to the space environment of the actual RA pannus tissue, enhancing the communication between cells and thereby improving the accuracy and efficiency of drug detection. For example, Kim et al. showed that cells grown in 2D culture conditions exhibit different gene and protein expression from those observed *in vivo*. And it was confirmed that cell proliferation rates in 3D culture was higher than that in the 2D cell culture because they mimic cell-to-cell interaction. They also found that there was a different anticancer effect between these two models. The drug effect in the 2D cell culture model is exaggerated, which explains why immunotherapies have shown excellent efficacy in research studies but not in clinical studies and patients (Kim et al., 2019). Thus, in this study we constructed the 3D pannus model based on the co-culture system of MH7A and EA.hy 926 cells. Alginate and gelatin are natural biomaterials. Both of them have high printability and good biocompatibility with cells. In addition, micro-extrusion printing has been shown that they can print cell laden scaffold in a controllable way with high cell viability. Despite cells experience stress condition during printing, they still have good cell viability with hydrogel (Mandrycky et al., 2016; Panwar and Tan, 2016). To maintain sufficient mechanical strength, printed scaffold needs to crosslink with CaCl_2_ solution every 3 days. Our result showed that CaCl_2_ solution had a cytotoxic effect among EA.hy 926 cells, MH7A cells, and 1:1 co-culture mixture groups in 2D planar. Cell viability decreased to around 30% after calcium stimulation for 5 min at 24 h, but cell viability regulated to around half at 48 h. According to the manufacturer’s instruction, the recommendation time for crosslinking is 4–8 min, and we chose 5 min in order to hold the shape of the scaffold. As the concentration and ratio of gelatin and sodium alginate are confidential and there would be difference between 2D culture and 3D hydrogel scaffold, we further tested cell survival using calcein-AM/PI staining assay and measured cell proliferation using CCK-8 assay in 3D cell laden scaffold. The survival result showed that the viability of the co-culture mixture was around 80%. The proliferation rate of co-culture cells increased steadily from day 1 to day 7, which illustrates the gelatin/alginate bioink is biocompatible with cell growth. Earlier studies have demonstrated that cell viability and behavior in the scaffold is influenced by biomaterial type, material viscosity, printing speed, printing temperature and extrusion pressure (Zhao et al., 2015; Li et al., 2018). During our exploration on printing process, we noticed that too low temperature would lead to over coagulation of bioink. The bioink was so difficult to extrude that shear forces increased, resulting in cellular injury and death.

Compared with the morphology of MH7A cells and EA.hy 926 in 2D planar culture and 3D printed scaffold, we found that MH7A showed epithelioid and polygonal morphology and EA.hy 926 presented epithelioid morphology while they looked spheroids from day 1 to day 7. What’s more, after we measured the diameter of cellular spheroids on day 1, 3, 5 and 7, we observed that cells were assembled to be larger spheroid with time.

VEGF and ANG are considered fundamental in the formation of pannus. VEGF is one of the key regulators of angiogenesis as they are related to proliferation, migration and vascular tube formation (Marrelli et al., 2011). ANG acts later in the pathogenesis of pannus compared to VEGF. ANG is increased to form and increase blood vessel stability (Clavel et al., 2003). In order to verify the biological function of our 3D printed scaffold, we conducted ELISA assay to detect concentrations of VEGF and ANG protein. The results showed that the expression of VEGF and ANG in the co-culture and pannus group increased with days, but the co-culture group showed a more obvious effect. This may be because the cells in pannus group was induced by TNF-α before the model was constructed, but we did not provide an external inflammatory environment afterwards, resulting in the incomplete performance of the pannus characteristics. MTX has been showed that it could reduce VEGF content in CIA rat model to relieve angiogenesis (Chen et al., 2021). Hirata S also illustrated that MTX inhibited both basal and vascular endothelial cell growth factor-stimulated tritiated deoxyuridine (3H-UdR) incorporation into vascular endothelial cell in a dose-dependent manner (Hirata et al., 1989). So MTX was added after the model was printed, and the concentration of pro-angiogenic factors decreased compared with the pannus group for 7 days.

However, there are some differences between our 3D pannus scaffold and the actual pannus tissue. Here we only chose vascular endothelial cells and synovial fibroblast to mimic the cell composition of RA pathological pannus tissue instead of using all cell types. The pro-angiogenic factors *in vivo*, such as growth factors, hypoxia inducible factors, cytokines, chemokines, matrix metalloproteinase and adhesion molecules are also complex in deterioration of pannus. In this study, we constructed a 3D co-culture model for RA pannus tissue and provided a basic view of its biological characteristics. Further work on the comparison of pathological characteristics between pannus model of RA *in vitro* and clinical pannus specimens is needed, and improvements of this scaffold should be processed in the future.

## Conclusion

We report the construction of *in vitro* RA pannus co-culture model by applying 3D printing technique with EA.hy 926/MH7A and gelatin/alginate. The 3D pannus model showed a good cell viability and interaction to mimic the microenvironment of pannus *in vivo*. The concentration of VEGF and ANG protein in the supernatant of the 3D pannus model increased over time. In addition, adding MTX to the 3D pannus model can down-regulate the expression of pro-angiogenic factors. Further studies are required to develop more details to construct the platform for drug screening, but this study may offer a basic view of 3D printed pannus model in drug screening application.

## Data Availability

The raw data supporting the conclusion of this article will be made available by the authors, without undue reservation.
