# Supplementary material for: A Three-Dimensional Co-Culture Model for Rheumatoid Arthritis Pannus Tissue
Source: Front Bioeng Biotechnol. 2021 Nov 12;9:764212. doi: 10.3389/fbioe.2021.764212 (PMC8638776; doi:10.3389/fbioe.2021.764212)
Supplement: Supplementary file 1 [file DataSheet1.docx]

Supplementary Material

# Supplementary Figures

**
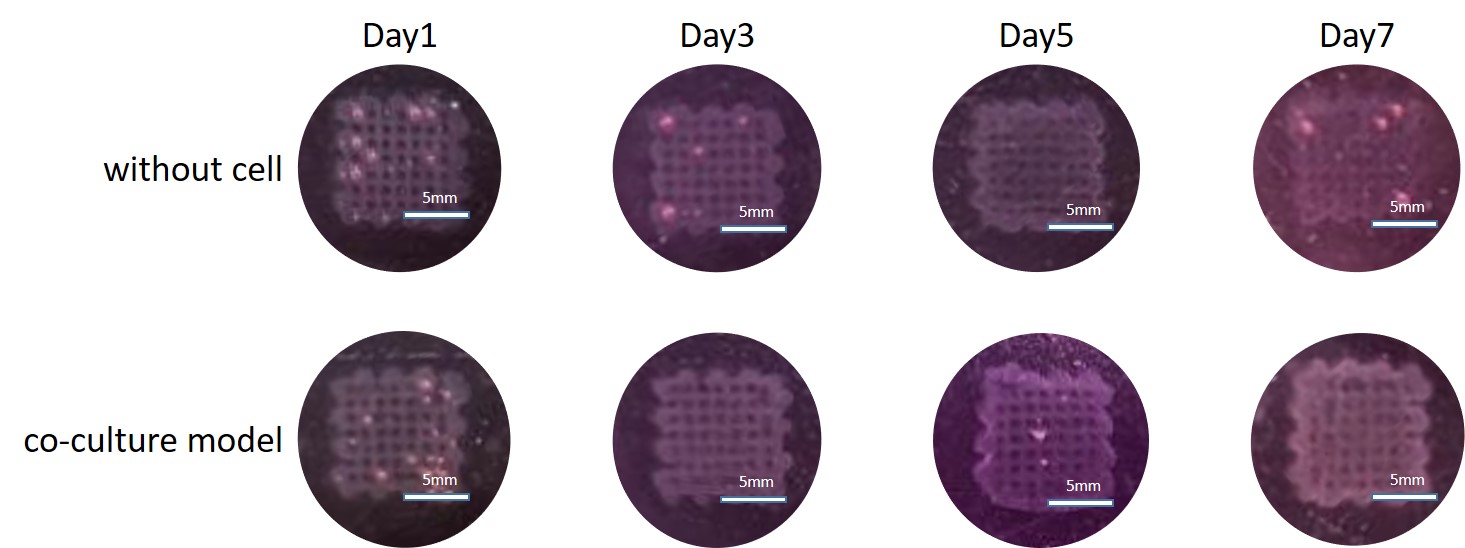
**

**Supplementary Figure 1.** Top view of 3D scaffolds on day 1, 3, 5, 7. Scale bar, 5mm.
